# Scaffold-Based (Matrigel™) 3D Culture Technique of Glioblastoma Recovers a Patient-like Immunosuppressive Phenotype

**DOI:** 10.3390/cells12141856

**Published:** 2023-07-14

**Authors:** Frank K. Braun, Tanja Rothhammer-Hampl, Julia Lorenz, Sandra Pohl, Ayse-Nur Menevse, Arabel Vollmann-Zwerenz, Elisabeth Bumes, Maren Büttner, Saida Zoubaa, Martin Proescholdt, Nils O. Schmidt, Peter Hau, Philipp Beckhove, Beate Winner, Markus J. Riemenschneider

**Affiliations:** 1 Department of Neuropathology, Regensburg University Hospital, 93053 Regensburg, Germany; 2 Division of Interventional Immunology, Leibniz Institute for Immunotherapy, 93053 Regensburg, Germany; 3 Department of Neurology and Wilhelm Sander-NeuroOncology Unit, Regensburg University Hospital, 93053 Regensburg, Germany; 4 Institute of Computational Biology, Helmholtz Center Munich, 85764 Munich, Germany; 5 Department of Neurosurgery, Regensburg University Hospital, 93053 Regensburg, Germany; martin.proescholdt@ukr.de (M.P.); nils-ole.schmidt@ukr.de (N.O.S.); 6 Department of Internal Medicine III, University Hospital Regensburg, 93053 Regensburg, Germany; 7 Department of Stem Cell Biology, Friedrich-Alexander-Universität (FAU) Erlangen-Nürnberg, 91054 Erlangen, Germany; 8 IZKF Junior Research Group 3 and BMBF Research Group Neuroscience, Interdisciplinary Center for Clinical Research, Friedrich-Alexander-Universität (FAU) Erlangen-Nürnberg, 91054 Erlangen, Germany

**Keywords:** brain cancer, tumor organoids, GBM, next-generation sequencing, single-cell RNA sequencing

## Abstract

Conventional 2D cultures are commonly used in cancer research though they come with limitations such as the lack of microenvironment or reduced cell heterogeneity. In this study, we investigated in what respect a scaffold-based (Matrigel™) 3D culture technique can ameliorate the limitations of 2D cultures. NGS-based bulk and single-cell sequencing of matched pairs of 2D and 3D models showed an altered transcription of key immune regulatory genes in around 36% of 3D models, indicating the reoccurrence of an immune suppressive phenotype. Changes included the presentation of different HLA surface molecules as well as cellular stressors. We also investigated the 3D tumor organoids in a co-culture setting with tumor-infiltrating lymphocytes (TILs). Of note, lymphocyte-mediated cell killing appeared less effective in clearing 3D models than their 2D counterparts. IFN-γ release, as well as live cell staining and proliferation analysis, pointed toward an elevated resistance of 3D models. In conclusion, we found that the scaffold-based (Matrigel™) 3D culture technique affects the transcriptional profile in a subset of GBM models. Thus, these models allow for depicting clinically relevant aspects of tumor-immune interaction, with the potential to explore immunotherapeutic approaches in an easily accessible in vitro system.

## 1. Introduction

Glioblastoma (GBM), IDH-wildtype (CNS WHO grade 4), is an aggressive and the most prevalent malignant primary brain tumor in adults, which presents with poor therapy response and prognosis. The 5-year survival rate for GBM patients is around 7%, with a median overall survival of 14.6 months [1,2,3]. State-of-the-art brain tumor classification employs a growing array of molecular markers in addition to histological features [2]. Genetic and epigenetic markers are also used to separate GBM into classic, proneural, and mesenchymal subtypes [4,5].

Despite advances in therapies for other cancer types, GBM remains difficult to treat. Unchanged since 2005, the standard of care for GBM patients is gross total resection followed by radiation therapy (XRT, external beam X-ray therapy) with concurrent and adjuvant chemotherapy with temozolomide (TMZ) [3,6,7]. Therapeutic obstacles are manifold, ranging from the large degree of tumor cell heterogeneity, and disseminated tumor growth to difficulties in drug administration through the blood–brain barrier or diminished uptake by the tumor cells [8,9]. In recent years, therapies targeting the immune status of tumor cells have been introduced with promising results for several diseases [10]. Key factors in effective tumor surveillance are, e.g., the MHC (major histocompatibility complex) class family with the expression of HLA (human leukocyte antigen) members [11,12]. In addition to inhibitory factors presented on tumor cell surfaces, shedding of receptors that bind cytotoxic ligands has been shown to interfere with immune surveillance by generating an immune suppressive environment [13,14].

Conventional in vitro cell culture models are recognized to not adequately represent many cancer entities. Various culture conditions such as stem cell or patient-derived cultures have been adopted to improve the situation. A remaining key problem with in vitro cultures is the lack of disease-specific microenvironments. The generation and use of multilayered 3-dimensional models is the latest approach aimed to address these limitations of 2D cultures [15,16,17]. Different 3D model protocols are reported, ranging from basic forms such as neurosphere cultures [18,19] to more complex setups such as organoids [15,17] and organotypic slice cultures [20]. Furthermore, 3D models with scaffolding (collagen/laminin, hyaluronic acid (HA)-rich hydrogel or poly(ethylene-glycol)-based hydrogels) and without scaffolding (hanging-drop) are reported [21]. Tumor organoids generated by following a Matrigel™-protocol benefit from a relative ease of use and scalability. Three-dimensional models can be generated using 3D-bioprinters, or incorporate additional tissue-specific cell types further improving the model system [22,23,24,25]. Other approaches to generate microenvironments are GLICOs (cerebral organoid glioma). In such models tumor cells are co-cultured with neuronal differentiated stem cells that provide a microenvironment [26,27].

In this study, we aimed to characterize scaffold-based (Matrigel™) 3D cultures and describe the potential of this tumor cell culture technique as a valid patient-derived in vitro model. Using a large number of primary human glioma cell cultures, we compared the 3D technique to a conventional 2D culturing approach in terms of the efficacy of generating models. We also compared matching pairs of 3D and 2D models by in-depth molecular characterization using NGS-based bulk and single-cell sequencing approaches. We identified a patient-like immunosuppressive phenotype in 3D models conveying resistance to lymphocyte-mediated tumor cell killing. As such, scaffold-based (Matrigel™) 3D culture could be a simple and valid model for predicting patient response to immunotherapy as well as testing new immune-based therapeutic approaches.

## 2. Materials and Methods

### 2.1. Patient Tissue and Model Generation

Freshly resected brain tumor tissue was collected in collaboration with the Department of Neurosurgery at the University Hospital Regensburg and processed into single-cell suspensions. All tumors were classified according to the WHO 2021 diagnostic criteria [2]. The following tumor types were included in the study: 43 glioblastomas, IDH-wildtype (CNS WHO grade 4), 8 astrocytomas, IDH-mutant (6 WHO grade 2 and 2 WHO grade 4 tumors), 7 oligodendrogliomas, IDH-mutant and 1p/19q-codeleted (2 WHO grade 2 and 5 WHO grade 3 tumors), 1 meningioma (CNS WHO grade 1), 1 paraganglioma (CNS WHO grade 1), 1 pleomorphic xanthoastrocytoma (CNS WHO grade 3), and 1 rare high-grade neuroepithelial tumor with MN1 alteration (HGNET-MN1).

Brain tumor sample acquisition, tumor cell enrichment protocols, and tumor-infiltrating lymphocyte isolation were approved by the Ethics Committee of the University Regensburg (No° 18-207-101 and No° 21-2393_1-101) and University Hospital Regensburg (No° 19-1454-101). Briefly, tissue was mechanically dissociated (surgical scissors) and screened using a 40 μm cell strainer. In order to further separate tumor cells from blood cells, the single-cell suspension was stacked on top of a biphasic gradient composed of LeukoSpin (pluriSelect, Leipzig, Germany, #60-00091-12)/PBMCSpin (pluriSelect, #60-00092-12), and samples were centrifuged (spin out) for 30 min at 1000 rpm without a break. Enriched tumor cell fractions were washed twice with PBS, and aliquots were used to start 2D and 3D (tumor organoid) cell cultures. Growth of patient-derived cultures was monitored daily over an extended period of time and passaged if needed. All cell cultures were maintained in RHB-A-based serum-free culture media (Takara, Kusatsu, Japan, #Y40001), supplemented with penicillin-streptomycin (Merck, Darmstadt, Germany, #P4333), epidermal growth factor (EGF; ReliaTech, Hamburg, NY, USA, #100-009, 20 ng/mL), and basic fibroblast growth factor (bFGF; ReliaTech, #300-003, 20 ng/mL) and incubated at 37 °C, 5% CO_2_ and 95% humidity. Brain tumor cell lines BTIC10, -13, and -18 had been generated from resected tissue of human IDH-wildtype glioblastomas (CNS WHO grade 4) patients, as previously reported [28].

### 2.2. Tumor Organoid Generation

Tumor cells were centrifuged (5 min, 1000 rpm) and resuspended in 20 μL RHB-A growth media and mixed with ice-cold 80 μL of Matrigel™ (Corning, Corning, NY, USA, #356230). Mixture was plated in drops of 5 μL or 10 μL on parafilm and placed for 45 min in a 37 °C incubator to solidify. Spheroids were transferred and kept for 24 h without agitation; afterward, cultures were placed on an orbital shaker (80 rpm) for continued culture [15].

### 2.3. GFP-Labeling of Cell Models

The introduction of GFP in patient-derived cell models was performed using lentiviral particles (Amsbio, Abingdon, UK, LVP436) following the manufacturer’s recommendations. Resistance to Blasticidin S (CarlRoth, Karlsruhe, Germany, #CP14.2) was used for selection, and GFP expression was monitored by immunofluorescence microscopy and flow cytometry.

### 2.4. Generation of Tumor-Infiltrating Lymphocytes (TILs) and Flu-Antigen-Specific CD8+ T Cells (FluTC)

For the generation of TILs, freshly resected glioblastoma tissue was cut into smaller pieces and enzymatically digested in media supplemented with 400 μg/mL DNase I (Merck, #11284932001) and 400 U/mL collagenase type IV (Worthington, Columbus, OH, USA, #LS004209) for 2 h at 37 °C, 5% CO_2_. The digested tumor was then filtered through a 100 μm filter, centrifuged at 1400 rpm for 10 min, and co-cultured with 2 × 10^7^ irradiated allogeneic PBMCs from 3 different donors in 20 mL of expansion media supplemented with 30 ng/mL anti-CD3, 3000 U/mL IL-2 and 0.38 μg/mL Amphotericin B (Gibco, #15290-26) for 14 days. On days 5, 7, and 11, IL-2 was replenished with fresh expansion media [29]. On day 14, cells were collected, characterized by flow cytometry, and frozen in aliquots of 5 × 10^6^ in freezing media A (60% AB serum and 40% RPMI1640) and B (80% AB serum and 20% DMSO). FluTC was generated as previously described [30].

### 2.5. Real-Time Live-Cell Imaging Assay for 2D-3D Co-Cultures

For 2D cultures, stable GFP-expressing BTIC10, BTIC133, and BTIC129 glioblastoma tumor cells were seeded in 96-U bottom plate (5000–30,000 cells/well in 200 μL) 1 day prior to co-culture. Plates were pre-coated with a solution of 12 mg/mL Poly-(methacrylsäure-2-hydroxyethylester) (poly-HEMA; Sigma Aldrich, Saint Louis, MO, USA) dissolved in 95% ethanol. For co-cultures of HLA-A2-positive BTIC10, tumor cells were pulsed with 0.01 μg/mL flu-peptide for 1h. After pulsing, flu-peptide-containing media was removed, and tumor cells were co-cultured with FluTC for 3 days. For co-cultures of BTIC133 and BTIC129, TILs were pre-activated for 1 day by stimulating with plate-bound 4 μg/mL anti-CD3 antibody (Clone: OKT3, eBioscience, San Diego, CA, USA, #14-0037-82) and soluble 1 μg/mL of human anti-CD28 antibody (Clone: CD28.2, BioLegend, San Diego, CA, USA, #302902). Both activated TILs and FluTC were labeled with CellTrace™ Far Red (Invitrogen, Waltham, MA, USA, #C34564) according to the manufacturer’s instructions prior to co-culture. Subsequently, co-cultures were imaged for 3 days using Incucyte^®^ SX5 live cell imager (Sartorius, Göttingen, Germany) for the indicated time points at a 4× magnification (1 image/well/3h). Tumor cell and T cell areas were quantified with the Incucyte^®^ SX5 2020B (Sartorius) software by generating a top-hat filter-based mask for the calculation of the green (GFP-expressing tumor cells) or red (T cells), respectively. Tumor cells or T cells-only wells were used as controls. After 3 days of co-culture, supernatants of T cell tumor cell co-cultures were analyzed for levels of IFN-γ (Human IFN-γ ELISA Set, BD OptEIA, San Diego, CA, USA, #555142) according to the manufacturer ’s instructions.

### 2.6. DNA/RNA Isolation

Isolation of DNA and RNA used the AllPrep DNA/RNA kit (Qiagen, Hilden, Germany, #80004) following the manufacturer’s instructions. DNA/RNA samples were quantified using NanoDrop2000 (ThermoScientific, Waltham, MA, USA).

### 2.7. “Bulk” RNA Sequencing

Libraries were generated using the Illumina stranded mRNA Library Prep kit (Illumina, San Diego, CA, USA, #20040529) with RNA input ranging from 50 to 200 ng. Library quality was analyzed using a TapeStation or Bioanalyzer instrument. Sample preparation was performed according to the manufacturer’s instructions. Libraries were equimolarly pooled and sequenced. NGS data analysis was performed with freely available, customizable tools on a Linux-based workstation. BCL files were demultiplexed and converted into .fastq files with the bcl2fastq tool from Illumina Version 1.8.4. Adaptor trimming was performed with the cutadapt tool [31], and quality checks in the standard fastq.gz format were performed with fastQC [32]. The resulting fastq files were aligned to a reference genome (GRCh38.87 v102) using HiSat2 v2.0.4 [33] and further processed (sorted and indexed) with SAMtools 1.2 [34]. All unique hits were extracted using featureCounts v1.5.0 [35], and a count table was generated. Analysis was performed with R v4.2.1, and differential gene expression was calculated using DESeq2 [36] and adaptive shrinkage estimator from the ashr package [37]. Volcano plots and heatmaps were generated with the Enhanced Volcano and pheatmap v1.0.12 R packages [38]. Gene enrichment analyses used DESeq2 normalized expression values with the GSEA module v20.4.0 [39].

### 2.8. Single-Cell RNA Sequencing

For single-cell RNA sequencing analysis of 2D and 3D models, tumor cells were harvested and processed into single-cell suspension followed by a separation of cells according to GFP expression using fluorescence-activated cell sorting (FACS). Libraries of each sample were generated from a total of 20,000 cells and processed following the 10xGenomics protocol (Chromium Next GEM Single Cell 3′ Kit v3.1, #1000269). A total of 10x 3′ gene expression libraries were pooled and sequenced by short-read sequencing on an Illumina NextSeq 500 system using a high-output 150-cycle reaction kit according to the manufacturer’s protocol with the following read lengths: read1 28 nt; i7 index 8 nt; and read2 130 nt. A total of 10x 5′ gene expression libraries were also pooled and sequenced with 10× recommended read lengths (read1 26 nt; i7 index 8 nt; and read2 132 nt) or with extended R1 protocol (read1 158 nt; i7 index 8 nt; no read 2). Sequencing output files were processed (putative doublets and empty droplets according to total UMIs/cell removed, number of genes/cell, and percent of mitochondrial gene expression) using STARsolo v2.7.9a [40] on a Linux-based workstation. The resulting output files (barcodes.tsv.gz, features.tsv.gz, and matrix.mtx.gz) were then analyzed using R v4.2.1 and the Seurat v4.0.6 package [41]. Cerebro was used to generate UMAP and Sankey plots [42]. The 2D and 3D samples of BTIC10 and BTIC18 consisted of 3273 cells, 1847 cells, 2325 cells, and 6447 cells, respectively. The clustering resolution was set to 1.5 for the analysis of 10 principal components (PC). Differential gene expression and comparative analyses on complex cell types for 2D and 3D samples were performed using the Seurat integration procedure. Gene set enrichment (GSEA) and over-representation analysis (ORA) of differentially expressed genes were performed using the Reactome analysis tool v82 and pathway browser v3.7 [43].

### 2.9. DNA Methylation

Bisulfite conversion of 500 ng DNA was performed using the EZ-DNA methylation gold kit (Zymo research, Irvine, CA, USA). Epigenome-wide methylation levels were determined using the Illumina Human MethylationEPIC Beadchip and Illumina HiScan array scanning systems (Illumina, San Diego, CA, USA). Datasets were analyzed using the DKFZ classifier platform (v11b4) [44]. Differential methylation analysis was performed using the R v4.2.1 and the ChAMP package [45].

### 2.10. Flow Cytometry Analysis

Surface expression of HLA-A/B/C (Biolegend, #311434) on tumor cells was investigated using AlexaFluor-647-labeled antibodies and flow cytometry. A corresponding IgG2a isotype control was used (Biolegend, #400203). Cell cycle analysis was performed by staining nuclei with DAPI (1 ng/μL) for at least 4 h. DNA fragmentation (subG1) was determined by analyzing the DNA content/distribution according to a well-established protocol [46].

## 3. Results

### 3.1. Generation and Characterization of Scaffold-Based (Matrigel™) 3D Cultures

Tumor organoid (TO) models were generated from patient tissue in order to assess the benefit of 3D techniques for successful model generation. Additional in-depth follow-up analyses compared 3D (with TME) and corresponding 2D GBM models with respect to altered cellular signaling pathways.

**Figure 1:**
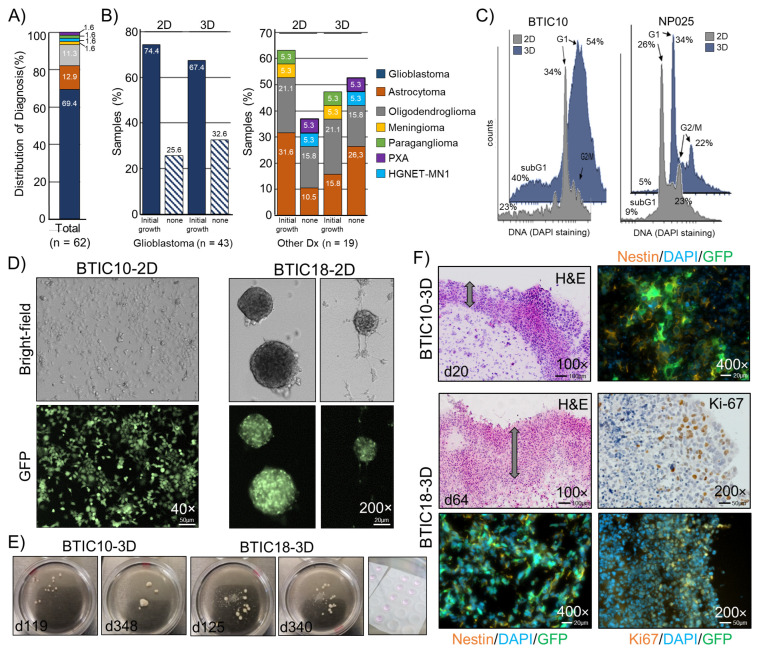
Establishment and characterization of patient-derived 3D cell culture models. (**A**) Distribution of diagnoses within the sample cohort in percent. (**B**) Samples with and without signs of growth for 2D and 3D cultures. Percent was calculated according to the total number of samples, glioblastoma (n = 43, left panel), and all other diagnoses (n = 19, right panel). (**C**) Representative histograms of the cell cycle analysis of two patient-derived GBM cell lines cultured in 2D and 3D. Percent cells are indicated. (**D**) Representative images of adherent or in suspension growing 2D cultures (passages 18 to 20). (**E**) 3D cultures shown at ~4 and ~12 months. Right image: Matrigel™ drops with mixed cells. (**F**) Images of H&E stained cryosections (8 μm) and sections stained for the stemness marker Nestin and the proliferation marker Ki-67 from areas of the cell dense edge region are shown, and constitutive GFP fluorescence is indicated. Nuclei counterstained with DAPI. The cell-rich outer layer is indicated by an arrow. Magnifications and scale bars are indicated.

For the model generation part, we collected and cultured tumor cells from surgically resected brain tumor tissue of 62 patients. The patient cohort had a median age of 58 years and a male/female ratio of 1.5. The collected samples represented a range of different brain tumor entities, with the majority of samples being diagnosed as glioblastoma, IDH-wildtype (GBM; 69.4%, n = 43), astrocytoma, IDH-mutant (12.9%, n = 8) or oligodendroglioma, IDH-mutant, and 1p/19q-codeleted (11.3%, n = 7). Other entities were only represented by single samples (Figure 1A). For GBM-derived cultures, we found that in 2D, 74.4% and in 3D, 67.4% of samples showed signs of growth, which indicates that both conditions are similarly effective in culturing GBM tumor cells (Figure 1B, left panel). This is, in general, also true for most of the other tumor entities. The only exception was astrocytoma, IDH-mutant CNS WHO grade 2, for which fewer 3D cultures were successful. Capacity to continued growth, as indicated by multiple passages, was found consistently in GBM samples for both culture conditions. For all other entities, the successful generation of models was very rare under both conditions.

Successful cell cultures displayed diverse growth patterns, ranging from growing strictly adherent to growing in suspension with varied-sized neurospheres (Figure 1D). We maintained individual 3D cultures for up to one year, which revealed that despite consuming growth media, the size and cell mass of TO are limited (Figure 1E). For the assessment of cell morphology, frozen sections (8 μm) of representative tumor organoids (3D) were generated and stained with hematoxylin and eosin (H&E). We observed that 3D models develop over time from an evenly distributed tumor cell suspension within the Matrigel™ drop to a multilayered spheroid. Key morphological features of these spheroids were a densely packed and vital outer layer with a sparsely populated core region showing signs of necrosis. Markers for proliferation (Ki-67) and stemness (Nestin) were analyzed by immunofluorescence staining, revealing expression of Ki-67 predominantly in the outer layer, whereas Nestin appeared widely expressed throughout the tumor organoid (Figure 1F).

Characterization of 2D (filled gray) and 3D (filled blue) models by DNA staining showed differences in cell cycle distribution (G1, S, G2/M) and cell viability (subG1) (Figure 1C). The increase in subG1 cell population (apoptotic cells) in 3D models is in line with the presence of necrotic cells in the core of 3D sections, as seen by H&E staining. However, for some GBM models, almost no differences in the cell cycle distribution were found when comparing 2D and 3D conditions. An epigenetic profiling was performed for sets of primary tumors (pt), 2D models, and 3D models of two GBM patients (BTIC18 and BTIC133). The models showed characteristic copy number variations (CNV) of disease-related genes (e.g., MDM4, EGFR, CDKN2A, CDK4, and MDM2) that were fairly consistent with the primary tumor (Figure S1A–D). Using an unsupervised clustering analysis of the methylation signature, we found that 2D and 3D cultures of the same model were largely similar in their methylation profile. However, there were larger differences when comparing the methylation profiles of the models to the corresponding primary tumor samples (Figure S1B,C). Of note, both primary tumors fell into the methylation class GBM, IDH-wildtype mesenchymal, while the culturing conditions changed the methylation class to either RTK I (BTIC18) or RTK II (BTIC133), most likely due to effects caused by the use of growth factor enriched cell culture media.

### 3.2. Transcriptome Characterization by Bulk RNA Sequencing

**Figure 2:**
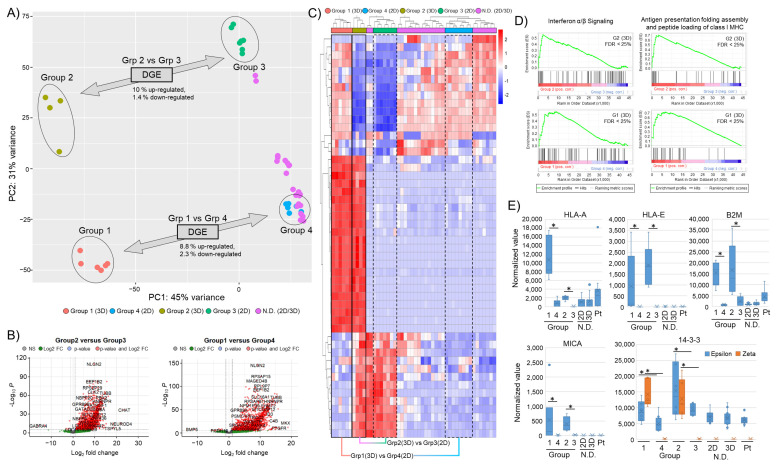
“Bulk” RNA sequencing of 2D and 3D cultured GBM models. (**A**) Principal component analysis of 11 GBM models. “Distinct” 3D (Group 1, Group 2) and corresponding 2D (Group 3, Group 4) samples are highlighted. Percent of differentially regulated genes of the groups are indicated. “Non-Distinct” (N.D.) 2D and 3D models are indicated. (**B**) Volcano plot of differentially regulated genes (2D versus 3D) with a log fold change >5 and *p*-value < 0.05. (**C**) Heat map of the top 50 most variable genes. Corresponding cultures are labeled 3D (solid box) and 2D (dotted line). (**D**) GSEA with enrichment plots for “Interferon α/β” (R-HSA-909733) signaling and “Antigen presentation, folding assembly and peptide loading of class I MHC” (R-HSA-983170) pathways using the Reactome Pathway Database (FDR score < 25%). (**E**) Normalized expression values for selected MHC class surface receptor genes (HLA-A, HLA-E), B2M, and stress-signaling markers (MICA and 14-3-3ε/14-3-3ζ). Asterisks indicate significant differences with *p*-value < 0.05.

In order to better understand the impact of 3D cell culture conditions on the transcription profile of tumor cells, we first applied a bulk RNA next-generation sequencing approach. Pairs of 11 GBM models (2D and 3D) were analyzed with corresponding patient material (Figure 2A). The analysis revealed that 3D samples from 4 (36%) of the 11 GBM models form distinctly different expression groups compared to corresponding 2D samples (Figure 1A–C, “distinct” models). For the other seven GBM models (“non-distinct” models, two of them were proneural and five mesenchymal), we did not find a similarly altered transcription profile (2D versus 3D). Within the four “distinct” models, we observed a further separation into two groups (Group 1, Group 2) that could be due to GBM-specific subclassification (proneural and mesenchymal). However, the proneural or mesenchymal transcriptional subtype did not inform on whether cultures were “distinct” or “non-distinct”.

Differential gene expression (DGE) analysis of “distinct” models revealed a gene upregulation of around 8.8–10% and less than 3% down-regulation (Figure 2B). A heat map of the top 50 most variable genes further highlights the separation of the identified groups (Figure 2C). To more thoroughly characterize the identified transcriptional changes, we performed a gene set enrichment analysis (GSEA). Here, we found that the differentially expressed genes in 3D cultured samples with a distinct expression profile were associated with immune regulatory mechanisms such as antigen processing and presentation or interferon signaling (Figure 2D). This points to a potential change in the immune phenotype of the “distinct” 3D cell cultures. mRNA levels of several immune regulatory (human leukocyte antigen (HLA)) and stress-signaling components (MICA/B, 14-3-3ε/ζ) were investigated and found to increase in the “distinct” 3D samples (Figure 2E). Interestingly, key immune surveillance molecules such as HLA-A, -B, and -C, as well as known immunosuppressive HLAs such as -E or -G, were elevated in 3D samples, also shown by surface staining using flow cytometry and immunofluorescence (Figure S2A,B). The increase in stress-related signaling molecules is in keeping with the observed morphology of 3D samples, possibly linked to nutrition and oxygenation gradients.

### 3.3. Altered Immunoregulatory Genes in “Distinct” 3D Cultures Are Also Found in Corresponding Primary Tumor Sample

**Figure 3:**
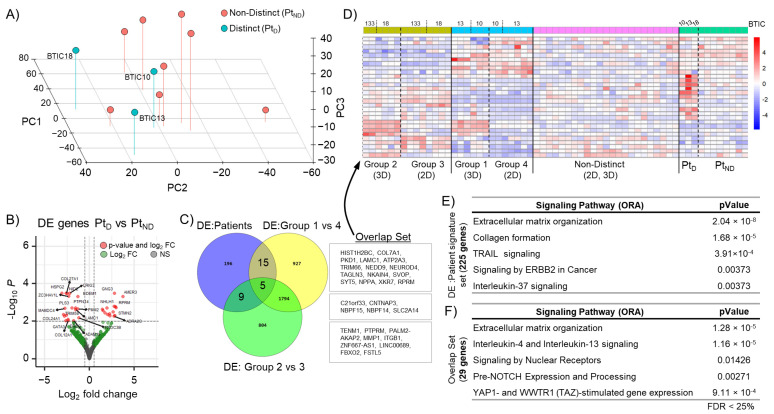
GBM patient signature gene set. (**A**) Analysis using the first 3 principal components of patient samples (n = 11), “distinct” (Pt_D_), and “non-distinct” (Pt_ND_) groups are indicated. (**B**) Volcano plot of differentially expressed genes between Pt_D_ vs. Pt_ND_ (Pt_D_ signature set) with a log fold change >2 and *p*-value < 0.05. (**C**) Venn diagram indicating an overlap in differentially expressed (DE) gene sets between Pt_D_ signature set and “distinct” 3D groups (Grp1vs4, Grp2vs3). (**D**) Heat map of overlap set (n = 29) gene expression in patient, 2D, and 3D samples. (**E**) Table for GSEA of differentially expressed genes of the Pt_D_ signature set (225 genes) and (**F**) of the overlapping gene set (29 genes).

Based on the 2D/3D bulk RNA sequencing data, we labeled patient samples as either “distinct” (Pt_D_) or “non-distinct” (Pt_ND_) (Figure 3A). A differential gene expression analysis revealed that 225 genes were altered between Pt_D_ and Pt_ND_ (Pt_D_ signature set; Figure 3B). In a further comparison of these genes with the genes differentially expressed in the “distinct” 3D groups (Grp1vs4, Grp2vs3), a common set of 29 genes (“Overlap Set”) was identified (Figure 3C). Expression of this overlap gene set (n = 29) was investigated in 2D, 3D, and patient samples and confirmed to be sufficient to separate the different groups (Figure 3D). An over-representation analysis (ORA) for both differentially expressed (DE) gene sets, the Pt_D_ signature set (n = 225) and the “overlap set” (n = 29) also revealed an association with immune regulatory and extracellular matrix signaling pathways (Figure 3E,F). A GSEA of the Pt_D_ signature set further revealed enrichment for programmed cell death-associated genes, such as “Regulation by cFLIP” and “Caspase-8 inhibition by regulated necrosis signaling” (Figure S3).

### 3.4. Single-Cell RNA Sequencing Reveals Formation of Clusters Enriched for Immune Regulatory Genes in “Distinct” 3D Cultures

**Figure 4:**
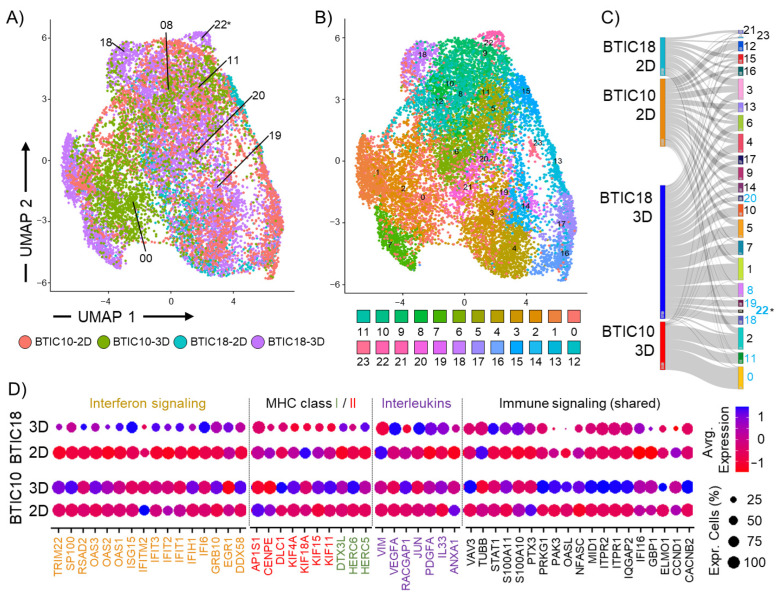
Single-cell RNA sequencing analysis of two GBM models. Shown are 2-dimensional reduction UMAP plots of an integrated analysis for 2 GBM models (BTIC10 and BTIC18) cultured in 2D or 3D for (**A**) models and culture conditions and (**B**) cell heterogeneity (cell clusters). Cell clusters of interest are indicated. (**C**) Sankey plot shows the contribution of models and culture conditions to identified cell clusters. Unique 3D cluster 22 is indicated (*), and 3D dominated clusters are highlighted (blue). (**D**) Expression of selected genes associated with immune regulatory signaling pathways (Interferon, MHC class I/II, Interleukins) is shown for 2D and 3D cultures of BTIC10 and BTIC18. Level of expression is indicated by circle size representing the percent of cells and with a color code indicating average expression (red = decrease, blue = increase).

Tumor heterogeneity, as represented by cells with different gene expression profiles, was investigated using single-cell RNA sequencing. For this approach, tumor cells from two GBM models with “distinct” 3D profiles, BTIC10 and BTIC18, were cultured in 2D and 3D and processed into single-cell RNA libraries using a 10xGenomics protocol. The sequencing datasets were analyzed using Seurat integrative analysis tools [41]. The analysis revealed a reshaping of 3D cultured models compared to corresponding 2D samples (Figure 4A,B). While largely similar in the cell cluster diversity (24 clusters), the number of cells representing specific phenotypes (cell clusters) varied. We found that six clusters were dominated by cells derived from 3D samples (cluster-0, -8, -11, -18, -19, and -20), indicating an expansion of these specific phenotypes under 3D culture conditions (Figure 4C). Expression of genes associated with immune regulatory signaling pathways (interferon (α/β, γ), interleukins, or MHC class I/II) from these cell clusters were analyzed in both models and culture conditions (2D and 3D) (Figure 4D). Both 3D models showed an altered expression of these genes compared to corresponding 2D models. A uniform 3D expression profile for both models was not identified, but this might be due to interpatient variability (Figure 4D).

Of note, we also found a unique 3D cell cluster (cluster-22) for BTIC18, which showed a gene enrichment for cell cycle, cellular response to stress, and interferon signaling.

### 3.5. Tumor-Immune Cell Co-Culture

**Figure 5:**
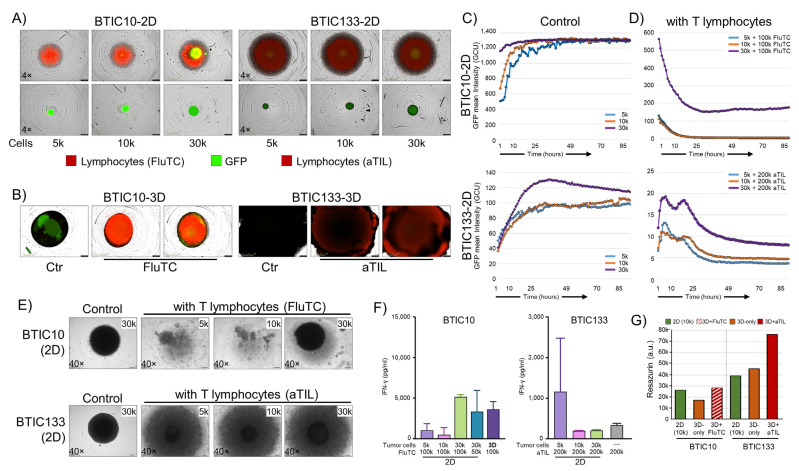
Functional analysis of modulated immunogenic phenotype. (**A**) Representative images of GFP-expressing BTIC10-2D and BTIC133-2D tumor cells (green) alone or in co-culture with activated tumor-infiltrating lymphocytes (red) are shown. Seeded numbers of tumor cells are indicated. BTIC10 was pulsed with flu-peptide and cultured with flu-specific CD8+ T cells (FluTC), and BTIC133 was cultured with autologous tumor-infiltrating lymphocytes (aTIL). Images shown represent day 3. (**B**) GBM models cultured in 3D, control cultures, and lymphocyte co-cultures are shown. (**C**,**D**) Cell proliferation according to GFP intensity for BTIC10-2D and BTIC133-2D. Seeding cell numbers are indicated. (**E**) Bright-field images of co-cultures on day 10. (**F**) Interferon-gamma (IFN-γ) release of BTIC10 and BTIC133 co-cultures at day 3, proving the functionality of immune cells (FluTC and aTILs). (**G**) Resazurin assay for 2D (10 k seeded cells, green), 3D (orange), and co-culture (FluTC, hatched red; autologous TIL, filled dark red) samples after 28 days of continued culture.

To better understand the impact of immune regulatory changes found in 3D samples, we used an immune-tumor co-culture system for 2D and 3D cultured tumor cells in a proof-of-concept approach. For BTIC133, autologous tumor-infiltrating lymphocytes (aTIL), and for BTIC10, flu-specific CD8+ T cells (FluTC) were used. GFP-expressing tumor cells (green) and labeled immune cells (red) were monitored for up to 90 h using an Incucyte^®^ SX5 live cell imaging system (Figure 5A,B). We found, for both GBM models cultured in 2D and in the absence of immune cells, a strong increase in proliferation was indicated by an increased GFP signal intensity (Figure 5C). The different 2D tumor cell seeding densities (5000–30,000 cells per well) reached a plateau after 25 h in culture. In this context, it should be kept in mind that quantifying the GFP of stacked cells due to culture in U-well plates is a known technical limitation. The GFP expression in BTIC133 is known to be less uniform compared to BTIC10, resulting in a larger data variance. Additionally, the large size of BTIC133-3D was difficult to image due to the technical limitations of the Incucyte^®^ SX5 imaging system.

When the same number of activated immune cells (aTILs or FluTC) were added to 2D and 3D tumor cell cultures only in the 2D samples, a rapid and substantial decrease in GFP expression was observed, indicating T cell-mediated cell killing (Figure 5D). For BTIC10-2D cultures, all but the highest cell number seeded (30k) were completely eliminated (0% GFP) within 16 h. The 30 k cell seeding condition plateaued at 27% GFP signal after 16 h with signs of an increase in GFP signal at the endpoint (90 h) (Figure 5D, top). It is noteworthy that it was not possible to re-establish cultures from these wells despite a continued culture for 10 days. In BTIC133, the autologous T cells appeared less cytotoxic as it took around 36 h to reach maximum tumor cell reduction (Figure 5D, bottom). However, similar to BTIC10, it was not possible to re-establish a culture from these wells either, indicating that despite the GFP signal, general cell viability was severely compromised.

In clear contrast to 2D samples, the corresponding 3D samples did not show similar signs of T cell-mediated cytotoxicity. Due to the technical limitation, this could be best assessed by observing the structural integrity of 3D spheroids (Figure 5B). As shown by H&E staining (Figure 1E) TOs consist of a small outer layer of cells essential for the overall structural integrity. Therefore, the elimination of cells should result in the disintegration and collapse of co-cultured 3D spheres, which was not observed. In addition, 3D samples were successfully maintained in culture for another 10 days, and culturing was only stopped for sample embedding.

In addition to monitoring immune cells, we investigated interferon γ (IFN-γ) release in the supernatant to confirm T-cell (aTILs, FluTC) activation (Figure 5F). In general, the levels of IFN-γ from aTIL co-cultures were much lower compared to FluTC co-cultures, which aligns with the observed reduced cytotoxicity of aTILs in BTIC133-2D co-cultures. Despite the lack of observed T cell-mediated cytotoxicity in 3D co-cultures, we found comparable levels of IFN-γ released in BTIC10-3D co-cultures. For BTIC133-3D, the levels of IFN-γ were undetectable, even though ‘aTILs only’ showed IFN-γ secretion (Figure 5F). Thus, in combination with the cytotoxic effects on the 2D cell models, both groups of immune cells (FluTC and aTILs) can be considered functional. Whether the observed lack of IFN-γ secretion in BTIC133-3D cells is the result of T cell-inhibitory effects of the 3D microenvironment remains to be determined. We then used a metabolic activity indicator (Resazurin) to assess cell viability and cell numbers of 2D and 3D samples, as this could also be related to reduced T-cell effectiveness. We found that the 3D samples, despite their size, corresponded to the 10k 2D cultures in their metabolic activity (Figure 5G). Of note, 3D samples that have been exposed to immune cells (aTIL or FluTC) appeared to have an increase in cell numbers by proliferation or metabolic activity. This effect was particularly strong for BTIC133-3D. Thus, our data show that 3D cultured samples of two GBM models not only survive co-culture with activated cytotoxic T cells but show signs of enhanced proliferation or metabolic activity.

## 4. Discussion

Cancer research is elemental in the pursuit of a better understanding of the critical mechanisms linked to therapy resistance and drug efficacy. The recent WHO classification of tumors of the central nervous system (CNS) underscores the diversity of the different tumor entities and the need for tailored treatment approaches [2]. Particular new molecular techniques such as methylation analysis, gene expression profiles, single-cell sequencing, or gene-fusion panels allow for even more detailed identification and classification of unique groups within cancers [47]. In contrast to the complexity of diagnostic tools, the lack of adequate models is apparent and hampers the development of new treatment strategies. Improving in vitro models by introducing advanced cell culture techniques that better recapitulate brain cancer disease/subclass-specific features are urgently needed.

In this study, we aimed to investigate if a 3D cell culture technique could improve patient-derived model generation and if tumor cell-derived 3D models could provide disease-specific features for advanced testing. The culture of primary tumor tissue, as adherent or suspension in vitro culture, is practiced in many laboratories with a plethora of protocols. The emergence of more complex 3-dimensional culture techniques is seen as a promising new modeling approach [17,48,49]. Starting in vitro cultures from primary tumors is challenging, as many brain cancer entities and subgroups appear to lack the capacity to continue growth under conventional in vitro conditions. This also highlights the still limited understanding of the requirements needed for a successful model generation. We compared the culturing of surgically resected and processed brain tumor tissue in both conventional (2D) and Matrigel™-based 3-dimensional (3D) cultures [15]. For a large and diverse set of primary brain cancer samples, we found no clear benefit in the use of this Matrigel™-based 3D protocol compared to conventional 2D culture in terms of the successful generation of in vitro models. Innate factors in each tissue sample appear to be determinative. However, when 3D models from patient tissue were generated, suitable maintenance and recapitulation of tumor cell heterogeneity were reported [26,48], which was also the case in our culture collective. These data highlight the role of such 3D models in some aspects of cancer research. Nevertheless, access to fresh patient material limits the wider applicability of this approach.

A pivotal aspect of our study is the characterization of 3D brain cancer models. For the analysis of altered signaling pathways and tumor cell heterogeneity, we selected GBM cell models and maintained them as 3D and 2D cultures. The introduction of a 3D microenvironment, a critical feature of solid tumors, provides a level of complexity not present in conventional 2D cultures [17]. Within a tumor microenvironment (TME), signaling cues connect tumor cells with surrounding normal cells [13,14,21,50]. Modeling such complex signaling/interaction networks is, of course, difficult and still remains largely the domain of in vivo approaches [51]. However, steps toward more elaborate 3D protocols, e.g., by incorporating different cell types [21,27], show that modeling of critical aspects of the TME is feasible.

The tumor organoids in this study were generated using a Matrigel™-based scaffold protocol [15,48]; however, different approaches for 3D protocols have been reported as well, some with and others without scaffolding material [21]. Each protocol comes with benefits and limitations; the use of a Matrigel™-based approach offers relative ease of use and scalability. Often tumor organoids are cultured for a limited time only, whereas we aimed to also maintain long-term cultures, i.e., >1 year. The long-term approach provides a level of certainty that also rare morphological changes that develop protracted or over time could be detected. A characteristic feature of our 3D models was densely packed outer layers with a diminishing cell count toward the center. The core region featured necrotic and compromised cells, similar to tumors of GBM patients [52]. Other aspects, such as the formation of nutrition and oxygenation gradients, featured in many solid tumors, were also shown to be present in our 3D cultures but not in 2D cultures [48].

The in-depth molecular characterization of our 3D GBM models revealed that compared to 2D several models showed significantly altered mRNA expression patterns (“distinct” models). The observed cellular plasticity, as seen in the expansion of cell clusters (phenotypes) using single-cell sequencing of “distinct” 3D models, revealed a change in immune regulatory signaling pathways. The inability of effective tumor surveillance is a recognized challenge for GBM [13,14]. Three-dimensional models could be a tool to mimic and study this aspect of GBM. However, it was also evident that interpatient variations play a critical role in the response of models to the 3D culture condition, as we observed changes in only a subset of GBM models (4 out of 11, 36%). Members of the YHWA/14-3-3 family are part of the dynamic response to oxidative stress, which is an observed morphology also in our experiments [48]. Indeed, we found stress-induced mediators such as YWHA/14-3-3 members or MICA/B increased in our “distinct” 3D models. For YHWA/14-3-3 members, an essential role in the DDR1 signaling cascade, which is linked to radio-chemoresistance, has been shown [53]. This indicates that a complex microenvironment is generated in 3D models, eliciting signaling similar to patient tumor cells. Another important element in effective immune surveillance is the expression and presentation of major histocompatibility complex (MHC) proteins. Modulation of key elements of MHC signaling can result in the disguise of tumor cells and promote tumor proliferation [11]. MICA and MICB are homologous proteins of MHC class I molecules and are frequently and abundantly expressed on the surface of tumor cells and are reported to bind to the NK cell receptor NKG2D [54]. Furthermore, MHC class I consists of classical and non-classical HLA molecules. In our 3D samples, we found an upregulation of members of classical HLA class Ia molecules (HLA-A/B/C) that are widely expressed on different cell types and are crucial for CD8+ T cells as well as for NK cell-mediated antitumor effects [55]. We also found increased expression of some non-classical HLA class Ib molecules (HLA-E/F/G), which are tissue restricted and are more associated with the maternal immune response/tolerance during pregnancy [56]. HLA-E has been reported to have a role in the presentation of antigens to NK cells and was shown to mediate immune suppressive effects by blocking NK cell activity [57]. Though the changes in the “distinct” 3D samples are complex, they highlight the opportunities/potential of 3D model systems in studying immune regulatory signaling.

One strategy to investigate the interaction of normal and tumor cells is the co-culture system. As we found in our 3D GBM models, the concomitant expression of several pro- and anti-immune markers, predicting the immunogenicity of these models was difficult. We, therefore, devised a tumor-immune cell co-culture testing strategy. For our 3D in vitro models, we found a considerable resistance toward T cell-mediated cell death compared to 2D counterparts, indicating that the identified changes in immune regulatory pathways promote an immunosuppressive environment. Thus, these 3D models are more suitable for investigating tumor–immune cell interactions (ICI) in vitro than conventional 2D models. Although immunotherapies have so far not been successful in brain cancer, adequate new model systems hold the promise of addressing some of the reported shortcomings [11].

## 5. Conclusions

We found that a Matrigel™ scaffold-based 3D cell culture technique does not improve the brain tumor model generation rate. However, our analysis of 11 cell line-derived 3D GBM models with corresponding 2D counterparts identified a significantly altered transcription profile in a subset of models. Particularly noticeable was the expansion of a patient-like immunosuppressive phenotype in 3D GBM models, highlighting disease-specific aspects modeled only in 3D cultures. The observed resistance to T cell-mediated cytotoxicity further underscores the potential/suitability of 3D models for the development and testing of immune cell-based therapeutic approaches.

## Data Availability

The data presented in this study are available on request from the corresponding author. The data are not publicly available due to privacy reasons.

## Supplementary Materials

The following supporting information can be downloaded at: https://www.mdpi.com/article/10.3390/cells12141856/s1, Figure S1: Epigenetic profiling of GBM models. (A) Copy number variation (CNV) profiles of patient tissue, 2D and 3D samples of two GBM models (BTIC18 and BTIC133). Loss and amplification of disease-related key critical markers are indicated. (B) PCA plot for sample methylation is shown, and corresponding samples are colored. (C) Dendrogram of an unsupervised hierarchical clustering analysis indicating samples with similar methylation profiles. (D) Sample information with diagnosis according to the DKFZ classifier v11b4. Figure S2: HLA surface expression in 2D and 3D cultured GBM models. (A) Surface expression of HLA-A/B/C (open graphs) and isotype controls (filled graph) of 2D (open black graph) and 3D (open blue graph) cell culture models. (B) Shown are sections (8 μm) of 3D samples and cytospins of 2D cell cultures stained with hemalaun and eosin (H&E) or against HLA-E (indicated by brown coloring). Nuclei counterstaining using hemalaun (blue). Magnifications are indicated. Figure S3: GSEA of PtD gene set. Reactome.org analysis of the PtD gene set (n = 225) was performed. Highlighted in yellow are signaling pathways related.
