# Supplementary figures and images for: Scaffold-Based (Matrigel™) 3D Culture Technique of Glioblastoma Recovers a Patient-like Immunosuppressive Phenotype
Source: Cells. 2023 Jul 14;12(14):1856. doi: 10.3390/cells12141856 (PMC10378658; doi:10.3390/cells12141856)

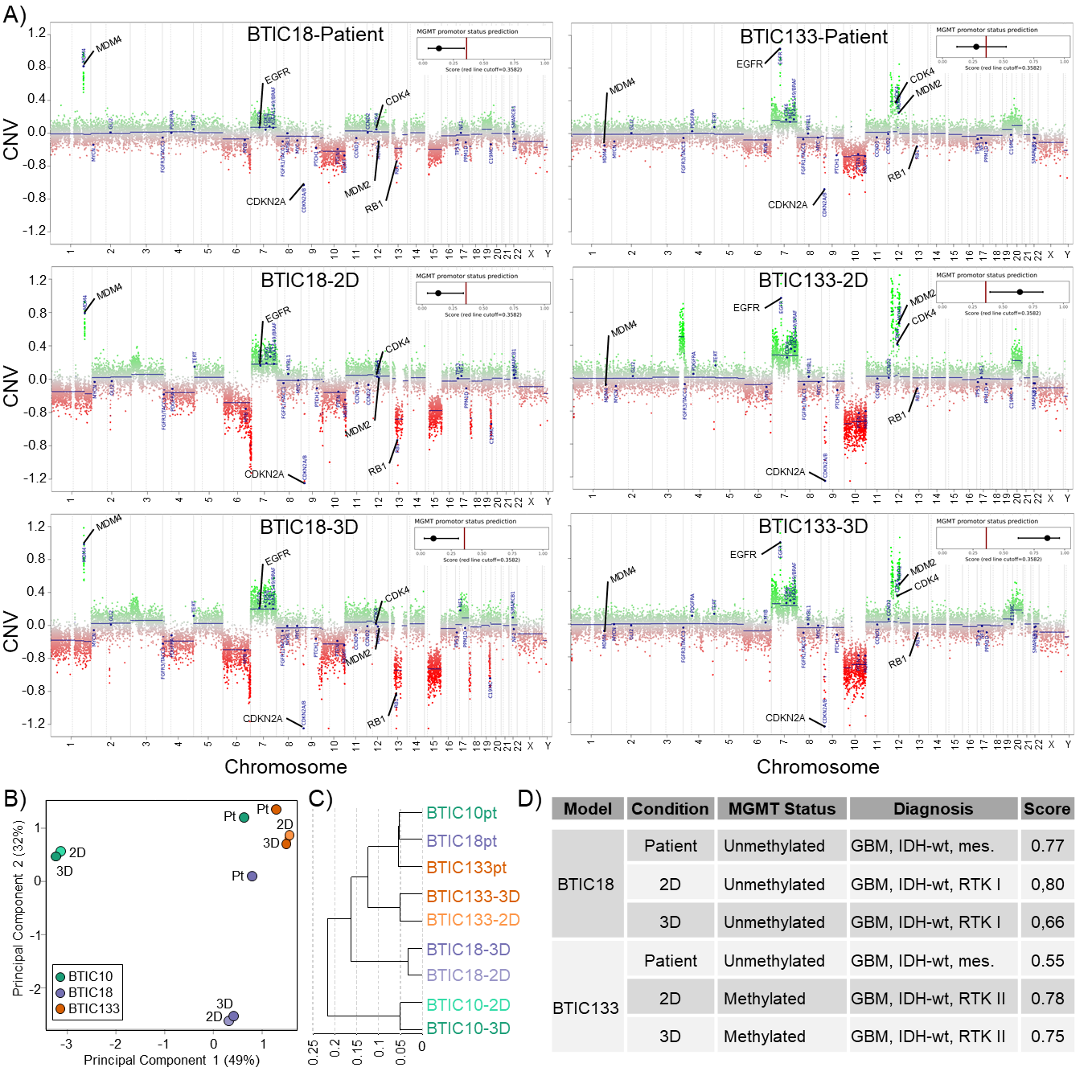

Supplement: Supplementary file 1 [file cells-12-01856-s001.zip › supp_Figure_1.tif]

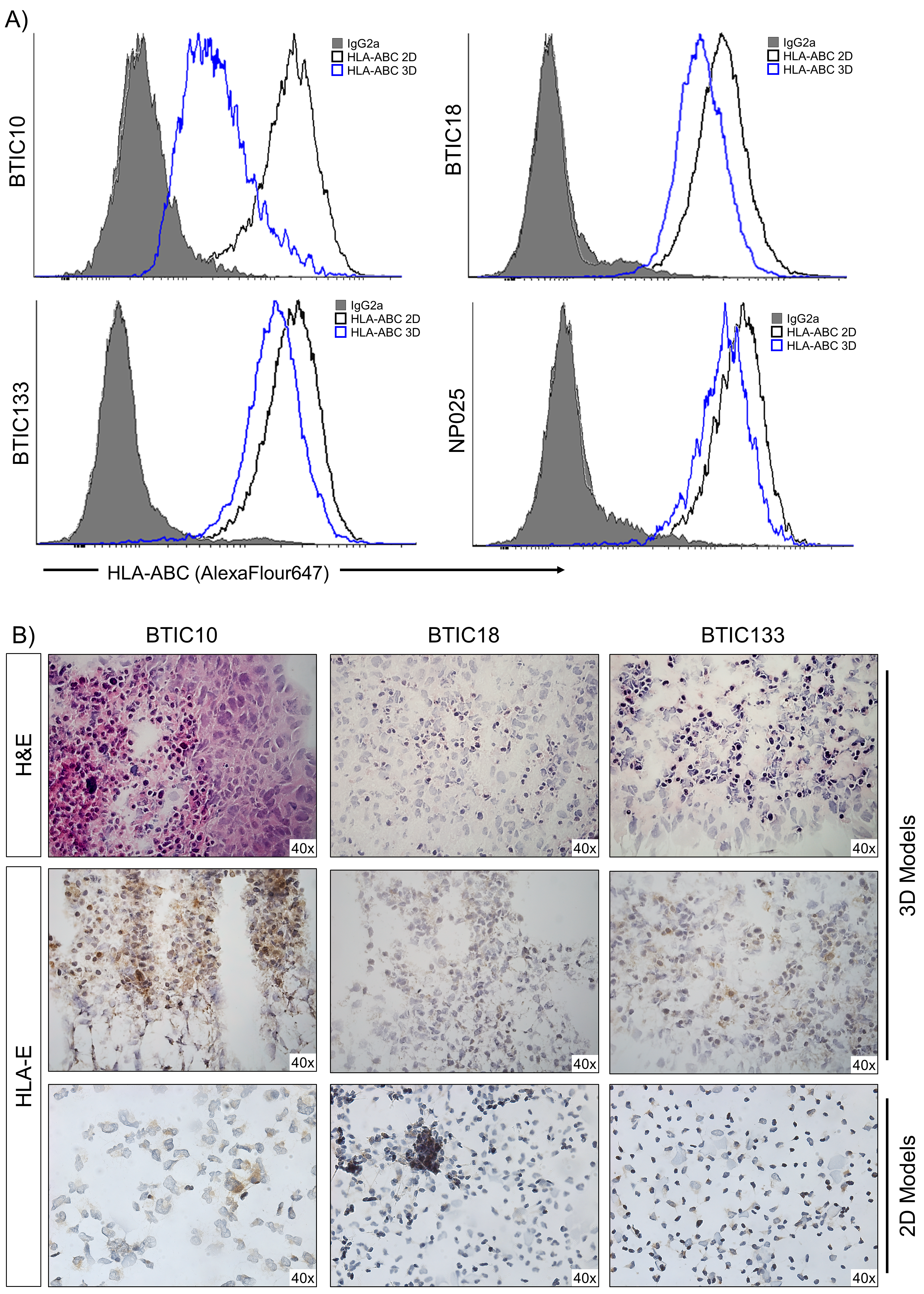

Supplement: Supplementary file 1 [file cells-12-01856-s001.zip › supp_Figure_2.tif]

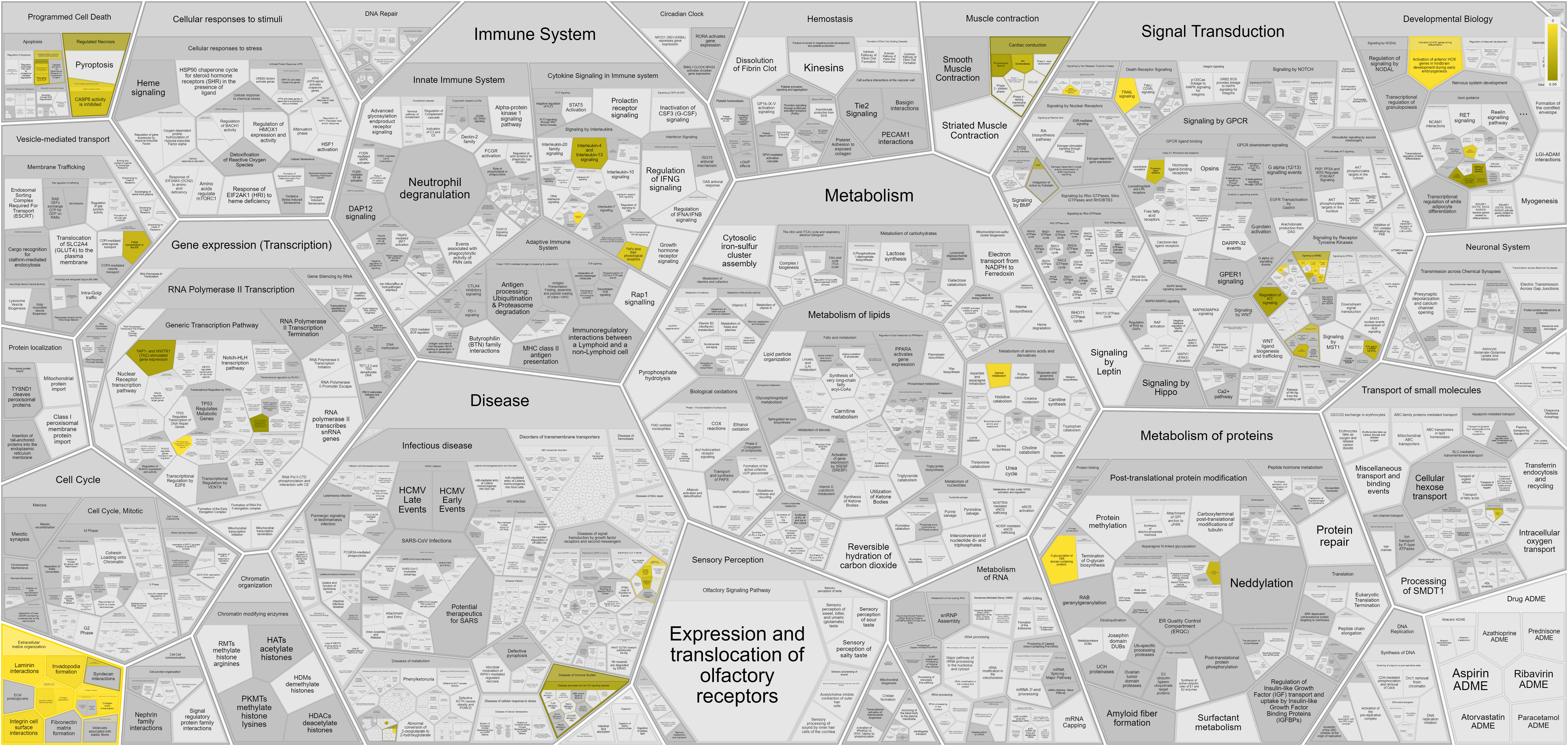

Supplement: Supplementary file 1 [file cells-12-01856-s001.zip › supp_Figure_3.tif]
